# Investigating the physicochemical properties, structural attributes, and molecular dynamics of organic–inorganic hybrid [NH_3_(CH_2_)_2_NH_3_]_2_CdBr_4_·2Br crystals

**DOI:** 10.1038/s41598-023-33192-1

**Published:** 2023-04-15

**Authors:** Ae Ran Lim

**Affiliations:** 1 Graduate School of Carbon Convergence Engineering, Jeonju University, Jeonju, 55069 Korea; 2 Department of Science Education, Jeonju University, Jeonju, 55069 Korea

**Keywords:** Materials science, Condensed-matter physics, Phase transitions and critical phenomena

## Abstract

An in-depth understanding of the physicochemical properties of the organic–inorganic hybrid [NH_3_(CH_2_)_2_NH_3_]_2_CdBr_6_ whose structure corresponds to the formulation [NH_3_(CH_2_)_2_NH_3_]_2_CdBr_4_· 2Br is essential for its application in batteries, supercapacitors, and fuel cells. Therefore, this study aimed to determine the crystal structure, phase transition, structural geometry, and molecular dynamics of these complexes. Considering its importance, a single crystal of [NH_3_(CH_2_)_2_NH_3_]_2_CdBr_6_ was grown; the crystal structure was found to be monoclinic. The phase transition temperatures were determined to be 443, 487, 517, and 529 K, and the crystal was thermally stable up to 580 K. Furthermore, the ^1^H, ^13^C, ^14^N, and ^113^Cd NMR chemical shifts caused by the local field surrounding the resonating nucleus of the cation and anion varied with increasing temperature, along with the surrounding environments of their atoms. In addition, ^1^H spin–lattice relaxation time T_1ρ_ and ^13^C T_1ρ_, which represent the energy transfer around the ^1^H and ^13^C atoms of the cation, respectively, varied significantly with temperature. Consequently, changes in the coordination geometry of Br around Cd in the CdBr_6_ anion and the coordination environment around N (in the cation) were associated with changes in the N–H···Br hydrogen bond. The structural geometry revealed critical information regarding their basic mechanism of organic–inorganic hybrid compounds.

## Introduction

Organic–inorganic perovskite-type compounds have been extensively studied in the field of photoelectronics and have been widely applied in systems such as solar cells and light-emitting devices^1–6^. The physicochemical properties and structural phase transitions of organic–inorganic compounds are related to their structure and the interactions between cations and anions^7^. Recent advances in the development of organic–inorganic hybrid compound-based solar cells have increased the demand for characterization of the dynamics and structures of their various constituents in relation to their potential impact^8^. In this study, the aforementoined properties were elucidated.

Organic–inorganic compounds based on zero- and two-dimensional perovskites [NH_3_(CH_2_)_*n*_NH_3_]*BX*_4_ (*n* = 2, 3, ∙∙∙; *B* = ^55^Mn, ^59^Co, ^63^Cu, ^65^Zn, ^113^Cd; *X* = Cl, Br)^9–17^ and [C_*n*_H_2*n*+1_NH_3_]_2_*BX*_4_^12, 18–20^ are of interest owing to their high thermal stability and broad application scope. Studies on [NH_3_(CH_2_)_*n*_NH_3_]*BX*_2_*X*_2_', containing different halogen ions, were reported by Abdel-Aal et al.^21–23^. An interesting group of hybrid compounds include perovskite-type layered compounds containing cations and layered metal-halogen anions. The physical properties of these compounds are attributed to the N‒H···*X* hydrogen bonds, between their cations and anions^13, 14, 24–26^. The structural flexibility and nonlinear optical properties of these perovskites are attributed to the organic material, whereas their thermal and mechanical properties are related to the inorganic material^27, 28^. These compounds are attractive because of their diverse crystal structures and phase transitions, which correlate with their cationic and anionic structural dynamics.

[NH_3_(CH_2_)_2_NH_3_]_2_CdBr_6_ crystals, namely [NH_3_(CH_2_)_2_NH_3_]_2_CdBr_4_·2Br, similar to the compounds mentioned above, were grown, and the structure of this single crystal and nuclear quadrupole resonance (NQR) studies of ^79^Br and ^81^Br were reported by Krishnan et al.^29^. The structure at 300 K was reported as monoclinic, space group *P2*_*1*_*/m* with the lattice constants *a* = 6.69 Å, *b* = 20.50 Å, *c* = 6.37 Å, β = 93.4°, and Z = 4. The two N(1) and N(2) atoms of [NH_3_(CH_2_)_2_NH_3_] cation were crystallographically inequivalent. Although the ^79,81^Br NQR spectrum experiment according to the temperature change and the crystal structure at 300 K was reported, the nuclear magnetic resonance (NMR) spectrum and spin–lattice relaxation time experiment for other nuclei were not performed.

In this study, single crystals of [NH_3_(CH_2_)_2_NH_3_]_2_CdBr_6_ whose structure corresponds to the formulation [NH_3_(CH_2_)_2_NH_3_]_2_CdBr_4_∙2Br were grown using an aqueous solution method, and their structures and phase transition temperatures (T_C_) were measured by the single crystal x-ray diffraction (SCXRD), powder x-ray diffraction (PXRD), and differential scanning calorimetry (DSC) experiments. To investigate the role of the [NH_3_(CH_2_)_2_NH_3_] cation in this single crystal, ^1^H magic-angle spinning (MAS) NMR, ^13^C MAS NMR, and ^14^N static NMR spectra were obtained as a function of temperature. In addition, the role of the CdBr_6_ anion was considered by the temperature-dependent chemical shift of ^113^Cd through ^113^Cd MAS NMR, and from this result, the N‒H∙∙∙Br hydrogen bond between the cation and anion was discussed. ^1^H T_1ρ_ and ^13^C T_1ρ_, which represent the energy transfer around the ^1^H and ^13^C atoms, was also considered. The results of the single-crystal structure and physicochemical properties predicted important information on the basic mechanism of organic–inorganic hybrid compounds.

## Methods

### Materials

Single crystals of [NH_3_(CH_2_)_2_NH_3_]_2_CdBr_6_, namely [NH_3_(CH_2_)_2_NH_3_]_2_CdBr_4_·2Br, were prepared from NH_2_(CH_2_)_2_NH_2_·2HBr (Aldrich, 98%) and CdBr_2_∙4H_2_O (Aldrich, 98%) with a ratio of 2:1 in ultra pure distilled water. The mixed substances were stirred and heated to obtain a homogeneous solution. Subsequently, the resulting solution was filtered, and transparent colorless single crystals were grown by slow evaporation in a thermostat at 300 K over three weeks. The crystals grew into rectangular shapes with dimensions of 5 × 5 × 1 mm.

### Characterization

The structures of the [NH_3_(CH_2_)_2_NH_3_]_2_CdBr_4_·2Br crystal at 250, 300, and 350 K were determined by SCXRD at the Korea Basic Science Institute (KBSI) Seoul Western Center. A crystal was mounted on a Bruker D8 Venture PHOTON III M14 diffractometer with a Mo–Ka radiation source. Data was collected and integrated using SMART APEX3 and SAINT (Bruker). The absorption was corrected using the multi-scan method implemented in SADABS. The structure was solved using direct methods and refined by full-matrix least-squares on F^2^ using SHELXTL^30^. All non-hydrogen atoms were refined anisotropically and hydrogen atoms were added to their geometrically ideal positions. The PXRD patterns were measured at several temperatures using an XRD system with a Mo–Ka radiation source.

DSC measurements were performed using a DSC instrument (TA Instruments, DSC 25) at a heating rate 10 °C/min, in the 200–553 K temperature range with the amount of the sample of 5.4 mg. The optical observations were measured by an optical polarizing microscope in the 300–573 K at heating stage of a Linkam THM-600. Thermal gravimetric analysis (TGA) was also performed with a heating speed 10 °C/min in the temperature between 300 and 873 K, under nitrogen gas.

The NMR spectra of the [NH_3_(CH_2_)_2_NH_3_]_2_CdBr_6_ crystals were measured using a solid-state 400 MHz NMR spectrometer (AVANCE III, Bruker) at the same facility, KBSI Western Seoul Center. To minimize the spinning sideband, samples in cylindrical zirconia rotors were spun at a spinning rate of 10 kHz for the MAS NMR measurements. Chemical shifts were referenced to adamantane for ^1^H and tetramethylsilane (TMS) for ^13^C, as standard materials for accurate NMR chemical shift measurements. MAS spin–lattice relaxation time T_1ρ_ values for ^1^H and ^13^C were performed using a π/2 − *τ* pulse by a spin-lock pulse of duration *τ*, and the π/2 pulse widths were measured using a previously reported method^28^. Further, static ^14^N NMR and ^113^Cd MAS NMR spectra of a [NH_3_(CH_2_)_2_NH_3_]_2_CdBr_6_ single crystal were measured at Larmor frequencies of 28.90 and 133.13 MHz, respectively, and the chemical shift was referenced with respect to NH_3_NO_3_ and CdCl_2_O_8_·6H_2_O as standard samples, respectively. ^14^N NMR spectra were obtained using a solid-state echo sequence.

## Experimental results

### Single-crystal XRD

**Figure 1:**
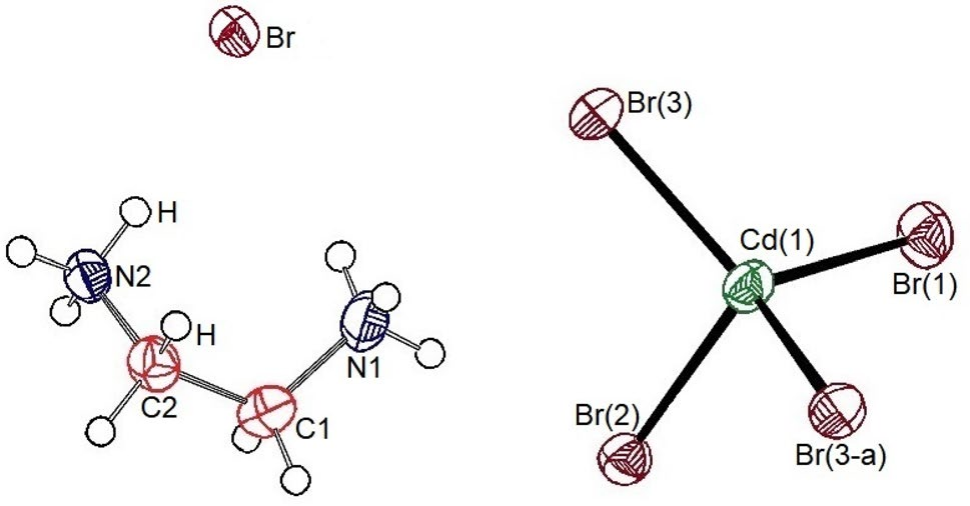
Thermal ellipsoid plot (50% probability) for [NH_3_(CH_2_)_2_NH_3_]_2_CdBr_4_·2Br structure at 300 K.

Single-crystal XRD results for the [NH_3_(CH_2_)_2_NH_3_]_2_CdBr_6_ crystals were obtained at 250, 300, and 350 K. At 300 K, the crystal grew a monoclinic system with *P2*_*1*_*/m* space group and lattice constants *a* = 6.393 (3) Å, *b* = 20.552 (8) Å, *c* = 6.710 (4) Å, *β* = 93.34° (3), and Z = 2. The thermal ellipsoids and atomic numbering for each atom are shown in Fig. 1, and the SCXRD data at 250, 300, and 350 K of the [NH_3_(CH_2_)_2_NH_3_]_2_CdBr_6_ crystal are shown in Table 1. Here, the lattice constants for the single crystals at 250 and 350 K were almost identical to those at 300 K. The two nitrogen atoms of the [NH_3_(CH_2_)_2_NH_3_] cation were crystallographically inequivalent, and the anion consists of a isolated tetrahedra [CdBr_4_]^2-^ and separate bromine ions [Br]^-^. The hydrogen atoms of each formula unit formed hydrogen bonds N − H∙∙∙Br between the anions and cations. The bond lengths and angles at 250, 300, and 350 K are listed in Table 2.

**Table 1:** Crystal data and structure refinement for [NH_3_(CH_2_)_2_NH_3_]_2_CdBr_4_∙2Br at 250 K, 300 K, and 350 K.

Temperature	250 K	300 K	350 K
Chemical formula	C_4_H_20_N_4_CdBr_6_	C_4_H_20_N_4_CdBr_6_	C_4_H_20_N_4_CdBr_6_
Weight	716.10	716.10	716.10
Crystal System	Monoclinic	Monoclinic	Monoclinic
Space group	P2_1_/m	P2_1_/m	P2_1_/m
*a* (Å)	6.387 (3)	6.393 (3)	6.3926 (2)
*b* (Å)	20.530 (9)	20.552 (8)	20.5822 (7)
*c* (Å)	6.699 (3)	6.710 (4)	6.7222 (2)
β (°)	93.473 (13)	93.34 (3)	93.4240 (10)
Z	2	2	2
V (Å^3^)	876.6 (6)	880.1 (7)	882.89 (5)
Radiation type	Mo-Kα	Mo-Kα	Mo-Kα
Wavelength (Å)	0.71073	0.71073	0.71073
Reflections collected	15,476	18,557	16,544
Independent reflections	2230 (*R*_int_ = 0.0432)	2240 (*R*_int_ = 0.0364)	2240 (*R*_int_ = 0.0411)
Goodness-of-fit on *F*^2^	1.041	1.090	1.045
Final *R* indices [I > 2sigma(I)]	*R*_1_ = 0.0203, *wR*_2_ = 0.0420	*R*_1_ = 0.0186, *wR*_2_ = 0.0419	*R*_1_ = 0.0223, *wR*_2_ = 0.0448
*R* indices (all data)	*R*_1_ = 0.0259, *wR*_2_ = 0.0437	*R*_1_ = 0.0210, *wR*_2_ = 0.0426	*R*_1_ = 0.0311, *wR*_2_ = 0.0477

**Table 2:** Bond lengths (Å) and bond-angles (°) for [NH_3_(CH_2_)_2_NH_3_]_2_CdBr_4_·2Br at 250 K, 300 K, and 350 K.

Temperature (K)	250 K	300 K	350 K
Cd-Br(1)	2.6130 (11)	2.6116 (15)	2.6109 (6)
Cd-Br(2)	2.7028 (11)	2.6981 (12)	2.6933 (6)
Cd-Br(3)	2.5737 (10)	2.5708 (9)	2.5685 (3)
Cd-Br(3-a)	2.5737 (10)	2.5707 (9)	2.5686 (3)
N(1)–C(1)	1.491 (4)	1.483 (4)	1.482 (5)
N(2)–C(2)	1.488 (3)	1.484 (3)	1.478 (4)
C(1)–C(2)	1.520 (4)	1.511 (4)	1.511 (5)
N–H	0.9000	0.9000	0.8900
C–H	0.9800	0.9800	0.9700
Br(3)-Cd-Br(3-a)	133.90 (3)	134.00 (3)	134.23 (2)
Br(1)-Cd-Br(3-a)	107.467 (14)	107.365 (16)	107.21 (11)
Br(3)-Cd-Br(1)	107.467 (13)	107.365 (16)	107.21 (11)
Br(2)-Cd-Br(3-a)	99.996 (13)	100.023 (15)	99.937 (12)
Br(1)-Cd-Br(2)	103.62 (2)	103.74 (3)	104.11 (2)
Br(3)-Cd-Br(2)	99.997 (13)	100.024 (15)	99.938 (12)

### Phase transition and powder XRD

**Figure 2:**
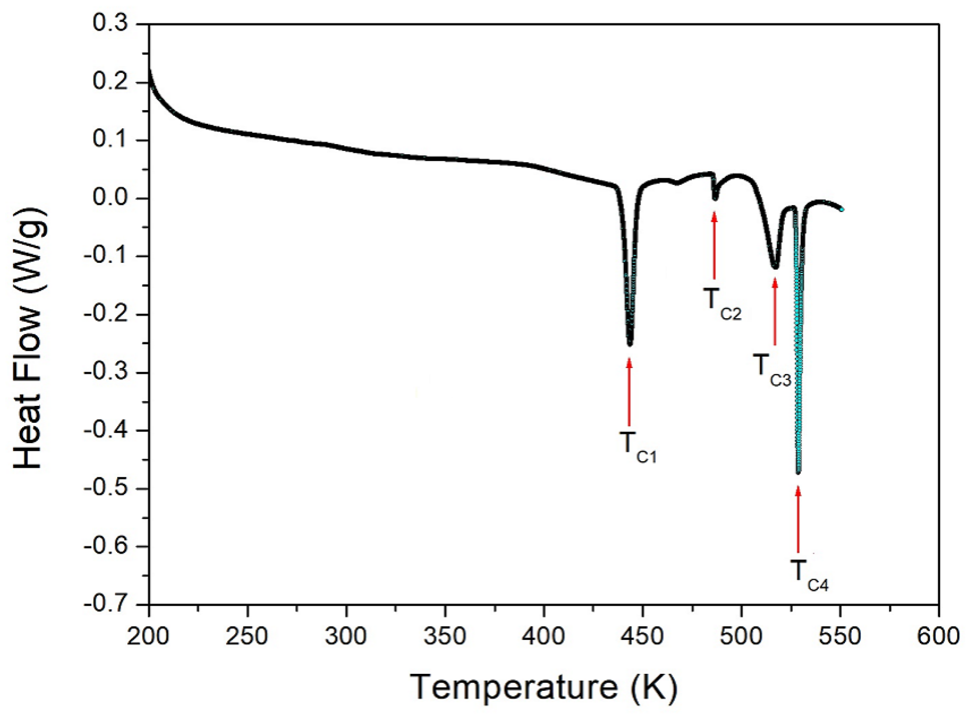
Differential scanning calorimetry curve of [NH_3_(CH_2_)_2_NH_3_]_2_CdBr_4_·2Br.

Figure 2 shows the results of the DSC experiment within the temperature range of 200–553 K with a heating rate of 10 °C/min. Three strong endothermic peaks at 443, 517, and 529 K, and one weak peak at 487 K were observed. The enthalpies of their peaks were 5.78, 0.28, 3.79, and 3.87 kJ/mol, respectively.

**Figure 3:**
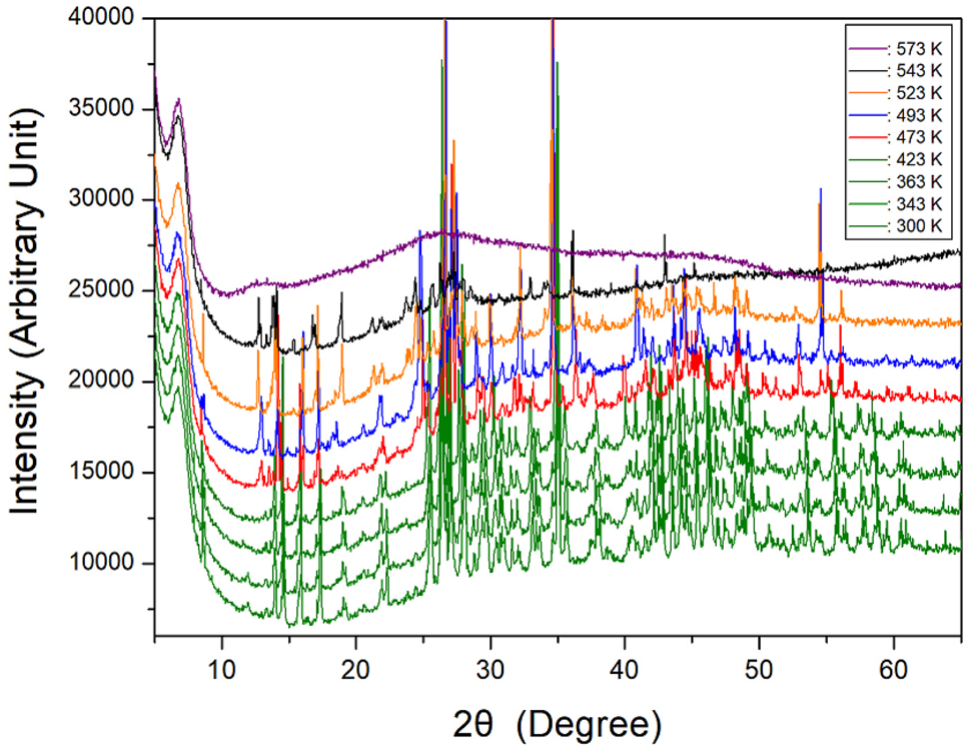
Powder x-ray diffraction patterns of [NH_3_(CH_2_)_2_NH_3_]_2_CdBr_4_·2Br at different temperatures.

To confirm whether the peaks in the DSC results shown in Fig. 2 are the phase transition temperature or melting point, a PXRD experiment was performed according to the temperature change. The PXRD patterns in the measuring range of 5–65° (2θ) are shown in Fig. 3 at several temperatures. The PXRD patterns below 443 K (olive) differ slightly from those recorded at 473 K (red); this difference is related to T_C1_ (= 443 K). Furthermore, the PXRD pattern recorded at 473 K varied from that recorded at 493 K (blue), and the PXRD pattern at 493 K differed from those obtained at 523 K (orange) and 543 K (black), related to T_C2_, T_C3_, and T_C4_ exhibiting a clear change in structure. The PXRD result was consistent with that of the DSC experiment, and it was determined that the four peaks corresponded to the phase transition temperatures. Finally, the pattern at 573 K was completely different from that at temperatures below 573 K, and crystallinity was not observed, indicating that it was the melting point.

Finally, the appearance of a single crystal was observed using an optical polarizing microscope. The crystal was colorless without significant change until the temperature was raised from 300 to 550 K. The surface of the single crystal began to melt at approximately 570 K. The phase transition and melting temperatures shown in the PXRD and optical polarizing microscope results are consistent with the temperatures shown in the endothermic peaks of the DSC curve. From the DSC, PXRD, and polarizing microscopy experiments, the phase transition and melting temperatures were determined as T_C1_ = 443 K, T_C2_ = 487 K, T_C3_ = 517 K, T_C4_ = 529 K, and T_m_ = 570 K.

### Thermal property

**Figure 4:**
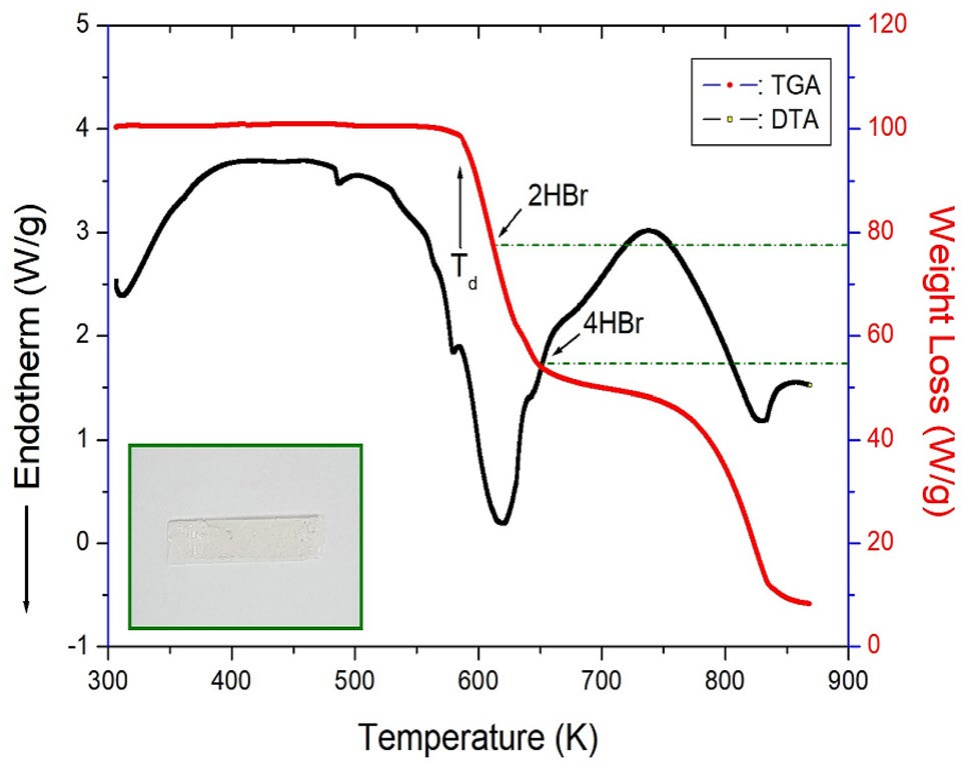
Thermogravimetry and differential thermal analysis curves of [NH_3_(CH_2_)_2_NH_3_]_2_CdBr_4_·2Br.

TGA curve was obtained, and the result is shown in Fig. 4. The TGA results revealed that this crystal is thermally stable up to 580 K. As shown in Fig. 4, the initial weight loss of [NH_3_(CH_2_)_2_NH_3_]_2_CdBr_6_ began at 580 K, representing a partial decomposition temperature with a weight loss of 2%. In the TGA curve, [NH_3_(CH_2_)_2_NH_3_]_2_CdBr_6_ exhibited a two-stage decomposition at high temperatures. The initial weight loss (50%) occurred in the range of 600–650 K, and second-stage decomposition (80%) occurred in the range of 650–800 K due to the inorganic moieties. The amount remaining was calculated from the TGA data and chemical reactions. The weight loss of 45% at approximately 650 K is likely due to the decomposition of the 4HBr moiety. The weight loss of 62% was mainly attributed to organic decomposition. One endothermic peak at 486 K on the DTA curve was assigned to the phase transition temperature obtained from the DSC results.

### ^1^H and ^13^C chemical shifts and spin–lattice relaxation times

**Figure 5:**
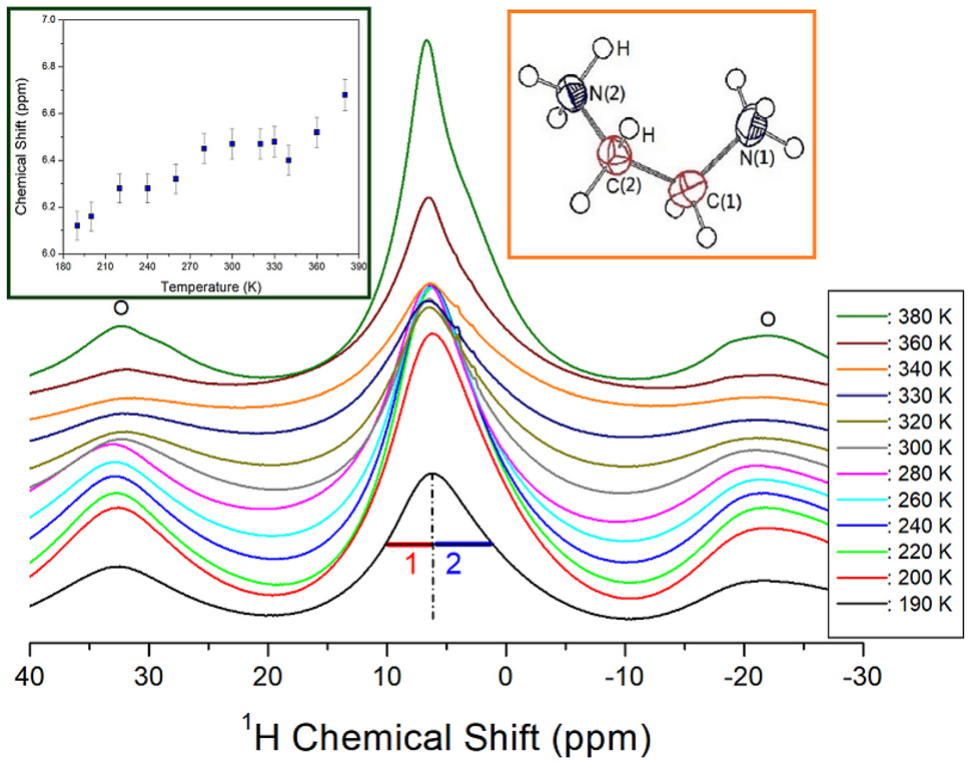
In-situ ^1^H MAS NMR chemical shifts for [NH_3_(CH_2_)_2_NH_3_]_2_CdBr_4_·2Br as a function of temperature.

The NMR chemical shifts for ^1^H in the [NH_3_(CH_2_)_2_NH_3_]_2_CdBr_6_ crystal were recorded by NMR spectroscopy using variable temperature analysis. At all temperatures, the resonance signals for NH_3_ and CH_2_ overlap, as shown in Fig. 5. The sidebands for the ^1^H signal are indicated by the open circles. Owing to the overlap of the ^1^H signals, the right side of the full width at half maximum (FWHM) appears to be slightly wider than the left side; the ^1^H chemical shift in NH_3_ on the left side and the ^1^H chemical shift in CH_2_ on the right side appear to overlap. The chemical shift and line width for ^1^H NMR spectrum at 300 K has 7.79 ppm and 7.85 ppm, respectively. The ^1^H chemical shifts showed a slight increase with temperature, hence, the coordination geometry around ^1^H changed with temperature.

**Figure 6:**
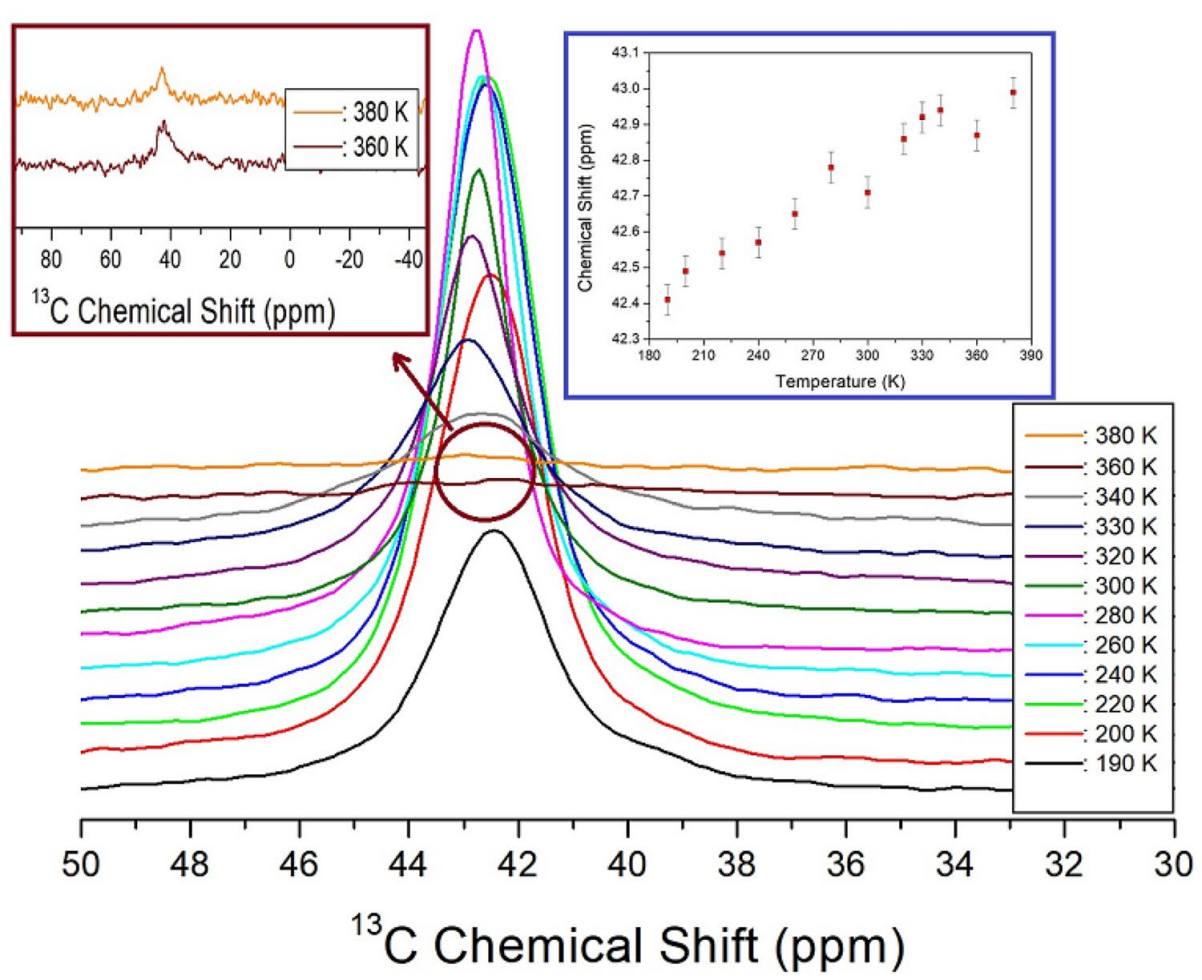
In-situ ^13^C MAS NMR chemical shifts for [NH_3_(CH_2_)_2_NH_3_]_2_CdBr_4_·2Br as a function of temperature (inset: ^13^C MAS NMR chemical shifts for [NH_3_(CH_2_)_2_NH_3_]_2_CdBr_4_·2Br as a function of temperature).

The ^13^C MAS NMR chemical shifts in the cation of [NH_3_(CH_2_)_2_NH_3_]_2_CdBr_6_ were also measured with increasing temperature, as shown in Fig. 6. The environments of the two CH_2_ groups, as shown in the cation structure of the crystal, were identical, thus only one resonance signal for CH_2_ was obtained. At 300 K, a ^13^C NMR chemical shift was observed at 47.71 ppm, and the line width was 1.75 ppm, which was very small compared to the ^1^H line width. The signal intensities at 360 K and 380 K are relatively small, and therefore, they are enlarged and shown again on the left side of Fig. 6. As the temperature increased, the chemical shifts increased without any anomalous change i.e., the coordination geometry of ^13^C continuously changed with temperature.

**Figure 7:**
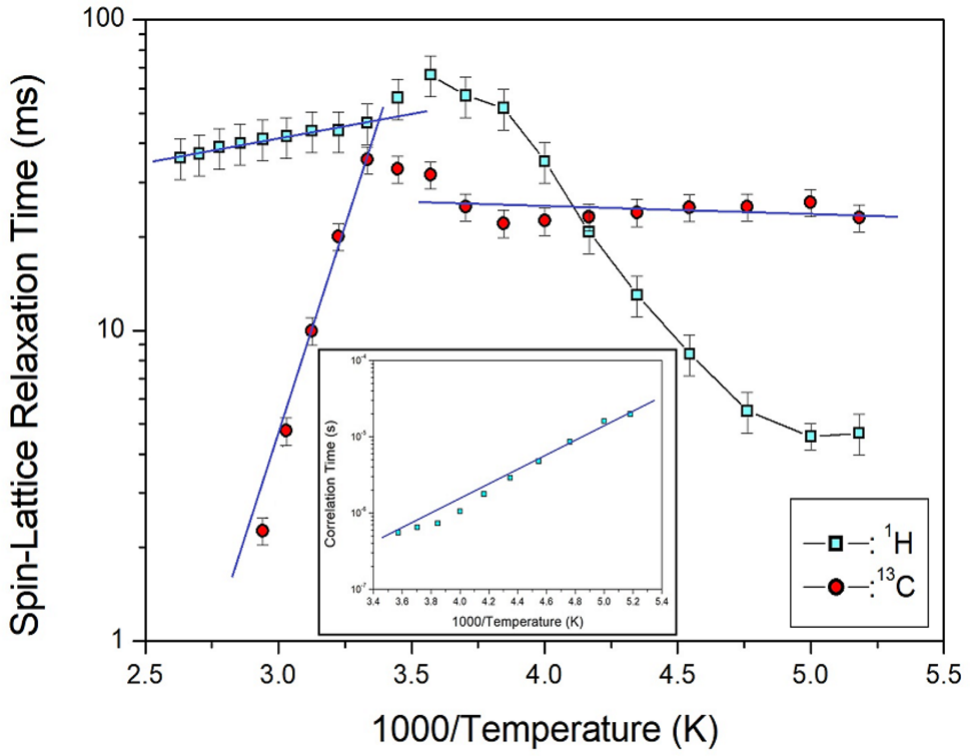
^1^H and ^13^C T_1ρ_ of [NH_3_(CH_2_)_2_NH_3_]_2_CdBr_4_·2Br as a function of 1000/temperature (inset: Correlation times for inverse temperature for ^1^H at low temperature; the lines represent activation energies).

To obtain the spin–lattice relaxation time T_1ρ_, the intensities of NMR spectrum for ^1^H and ^13^C NMR spectra were measured while increasing the delay times at a given temperature. The decay curves, according to the change in the signal intensities and delay times, are expressed as follows^31–34^:

1 $${\text{M}}\left( t \right) = {\text{M}}\left( 0 \right){\text{exp}}( - t/{\text{T}}_{{{1}\rho }} )$$

where M(*t*) is the resonance at time *t* and M(0) is the intensity of the resonance line at time *t* = 0. The magnetization decay curves were fitted using Eq. (1), thus, the T_1ρ_ values of ^1^H and ^13^C in [NH_3_(CH_2_)_2_NH_3_]_2_CdBr_6_ are shown in Fig. 7 as a function of the inverse temperature. The ^1^H T_1ρ_ values were strongly dependent on temperature. The ^1^H T_1ρ_ value increased rapidly from 5 to 70 ms between 193 and 280 K, and it decreases slightly with increasing temperature. T_1ρ_ values of ^1^H have minimum values at 200 K, indicating molecular motion according to the Bloembergen–Purcell–Pound (BPP) theory. It is clear that the T_1ρ_ minimum is attributable to the reorientational motion of NH_3_ and CH_2_. The experimental value of T_1ρ_ is expressed by the correlation time τ_C_ for the molecular motion by the BPP theory, where the T_1ρ_ value is given by^32, 34^:

2 $$\begin{aligned} \left( {{1}/{\text{T}}_{{{1}\rho }} } \right) &= {\text{C}}\left( {\gamma_{{\text{H}}}^{2} \cdot \gamma_{{\text{C}}}^{2} /r^{6} } \right)\{ {4}\tau_{{\text{C}}} /\left[ {{1 } + \left( {\omega_{1}^{2} \tau_{{\text{C}}}^{2} } \right)} \right] + \tau_{{\text{C}}} /\left[ {1 + \left( {\omega_{{\text{C}}} - \omega_{{\text{H}}} } \right)^{2} \tau_{{{\text{C2}}}} } \right] + {3}\tau_{{\text{C}}} /\left[ {1 + \omega_{{\text{C}}}^{2} \tau_{{\text{C}}}^{2} } \right] \hfill \\ &\quad + {6}\tau_{{\text{C}}} /[{1 } + \, (\omega_{{\text{C}}} + \omega_{{\text{H}}} )^{{2}} \tau_{{\text{C}}}^{{2}} ] \, + { 6}\tau_{{\text{C}}} /[{1 } + \omega_{{\text{H}}}^{{2}} \tau_{{\text{C}}}^{{2}} ]\} \hfill \\ \end{aligned}$$

Here, C is a constant; γ_H_ and γ_C_ are the gyromagnetic ratios for protons and carbon, respectively; *r* is the distance of the C-H internucleus; ω_1_ is the spin-lock field; and ω_C_ and ω_H_ are the Larmor frequencies for carbon and protons, respectively. The data was analyzed assuming that T_1ρ_ was the minimum when ω_1_τ_C_ = 1, and the relationship between T_1ρ_ and the radio frequency power of the spin-lock pulse ω_1_ was applicable. Because the T_1ρ_ curves were found to exhibit minima, the coefficient C in Eq. (2) could be determined. Using this C, we calculated τ_C_ as a function of temperature. The local field fluctuation is governed by the thermal motion of protons and carbons, which are activated by thermal energy. The correlation time τ_C_ of motion is generally assumed to have Arrhenius dependence on the activation energy for motion and temperature^33, 34^

3 $$\tau_{{\text{C}}} = \tau_{{\text{C}}}^{{\text{o}}} {\text{exp}}( - {\text{E}}_{{\text{a}}} /{\text{k}}_{{\text{B}}} {\text{T}})$$

where E_a_ and k_B_ are the activation energy of the motions and Boltzmann constant, respectively. The magnitude of E_a_ depends on molecular dynamics. To determine the molecular dynamics, we considered τ_C_ on a logarithmic scale vs. 1000/T as shown inset of Fig. 7; it was found to be 18.24 ± 1.25 kJ/mol at low temperature.

In contrast, the ^13^C T_1ρ_ value, unlike ^1^H T_1ρ_, shows an almost constant value below 300 K, but the T_1ρ_ value shows a tendency to decrease rapidly at temperatures above 300 K. The behaviors of the T_1ρ_ for Arrhenius-type molecular motions with relaxation time are split into fast- and slow-motion parts. Fast motion is represented as ω_1_τ_C_ « 1, T_1ρ_^–1^ ~ exp(E_a_/k_B_T), and the slow motion as ω_1_τ_C_ » 1, T_1ρ_ ~ ω_1_^–2^ exp(–E_a_/k_B_T). Different limits were satisfied for ω_1_τ_C_ in each temperature range, separated by T = 300 K. The ^1^H T_1ρ_ and ^13^C T_1ρ_ values at high temperatures were in the slow-motion regime, whereas the ^13^C T_1ρ_ values at low temperatures were attributed to the fast-motion regime. As represented by the blue lines in Fig. 7, E_a _= 2.85 ± 0.16 kJ/mol for ^1^H at high temperature, and E_a _= 0.48 ± 0.29 kJ/mol for ^13^C at low temperature, whereas E_a _= 50.98 ± 3.33 kJ/mol for ^13^C at high temperature (see Table 3).

**Table 3:** Phase transition temperature, T_C_, decomposition temperature, T_d_, spin–lattice relaxation time, T_1ρ_, and activation energy, E_a_, and line width, ∆w for [NH_3_(CH_2_)_2_NH_3_]_2_CdBr_4_·2Br.

T_C_ (K)	443, 487, 517, 529
T_d_ (K)	580
T_1ρ_ (*ms*)	46.51 for ^1^H at 300 K
	35.41 for ^13^C at 300 K
E_a_ (kJ/mol)	18.24 ± 1.25 for ^1^H at low temp
	2.85 ± 0.16 for ^1^H at high temp
E_a_ (kJ/mol)	0.48 ± 0.29 for ^13^C at low temp
	50.98 ± 3.33 for ^13^C at high temp
∆w (ppm)	7.85 for ^1^H
	1.75 for ^13^C
	41.94 for ^14^N
	6.23 for ^113^Cd

### Static ^14^N NMR chemical shifts

**Figure 8:**
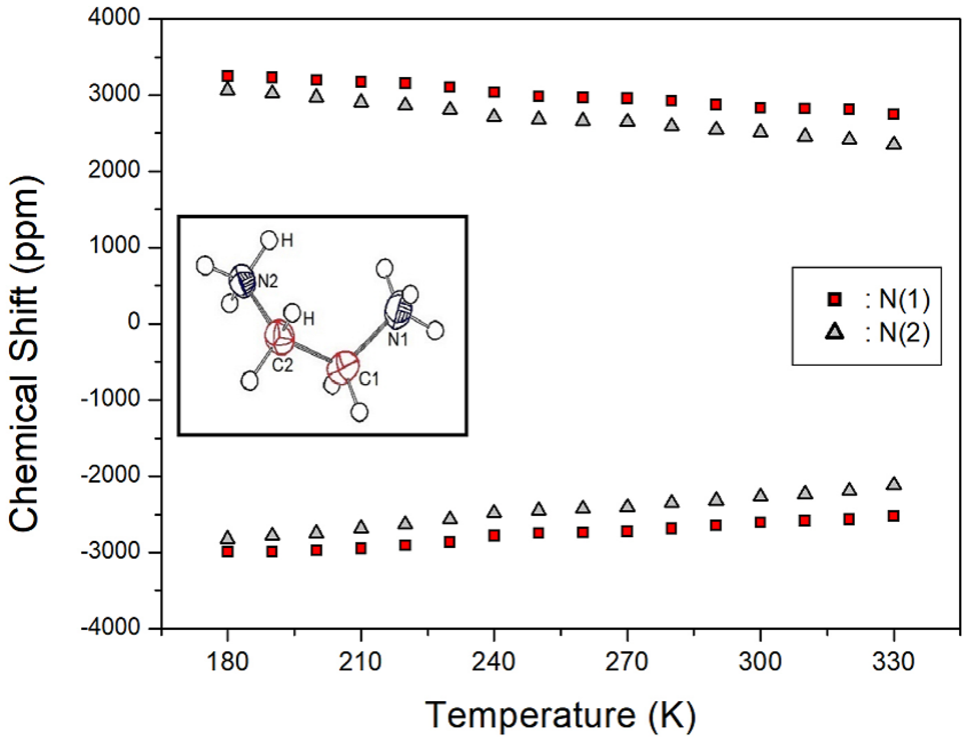
Static ^14^N NMR chemical shifts of [NH_3_(CH_2_)_2_NH_3_]_2_CdBr_4_·2Br single crystal as a function of temperature.

To investigate the structural geometry of ^14^N in the [NH_3_(CH_2_)_2_NH_3_] cation, static ^14^N NMR experiments were performed over a temperature range of 180–410 K using the solid-state echo method. The advantage of ^14^N NMR is its relatively small quadrupole coupling constant and the relatively simple structure of the NMR spectra by the spin quantum number (I = 1)^35, 36^. The applied magnetic field was measured in an arbitrary direction in the single crystal. The ^14^N NMR spectrum expected two ^14^N NMR signals because the spin number I = 1. Figure 8 shows two sets of signals, represented as squares and triangles, which are due to the crystallographically different N(1) and N(2) sites. At 300 K, the separation of the pair of lines was approximately 5405 and 4816 ppm for N(1) and N(2), respectively. As the temperature increased, the separation became narrower. This indicates that the quadrupole constant decreased as the temperature increased. N(1) is connected to Br(1), Br(2), and Br(3) by hydrogen bonds, whereas N(2) is connected to the surrounding Br via hydrogen bonds. The changes in the surrounding environment of N(1) and N(2), according to temperature change, were similar. In addition, this phenomenon was attributed to the changes in the structural coordinates and indicated the changes in atomic configurations around the ^14^N nuclei. The linewidth at 300 K was approximately 41.94 ppm, which was broader than those observed in the ^1^H and ^13^C NMR spectra. At temperatures above 330 K, it was difficult to obtain a signal because the intensity of the ^14^N signal was small and the line width was wide.

### ^113^Cd NMR chemical shifts

**Figure 9:**
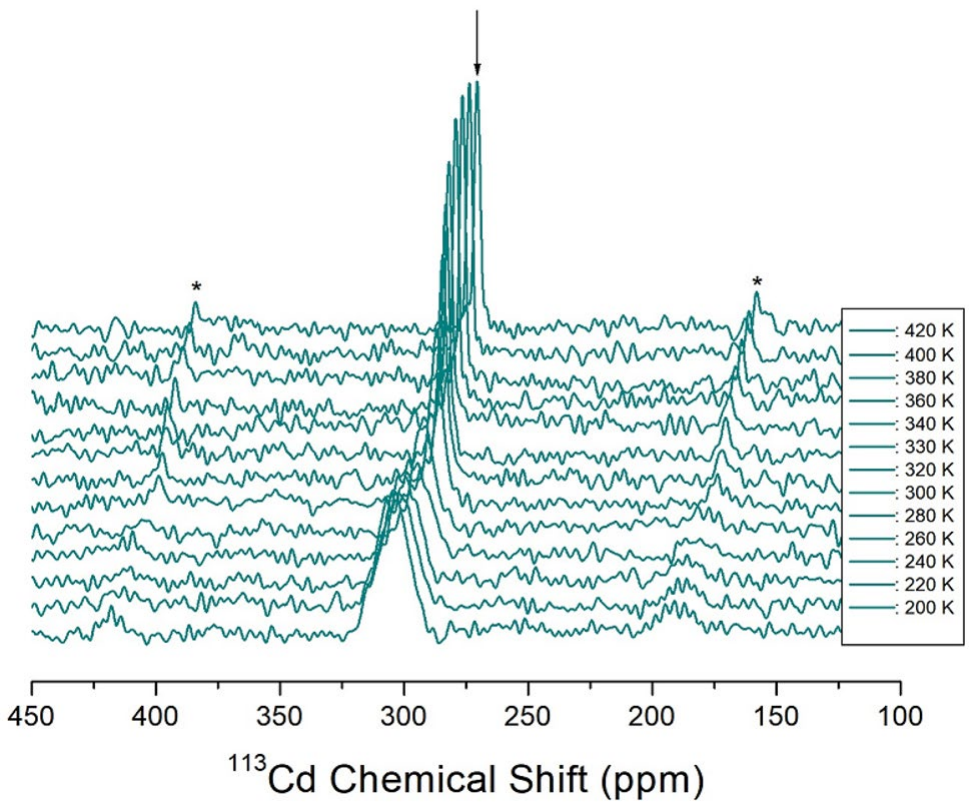
In-situ ^113^Cd MAS NMR chemical shifts of [NH_3_(CH_2_)_2_NH_3_]_2_CdBr_4_·2Br single crystal as a function of temperature.

**Figure 10:**
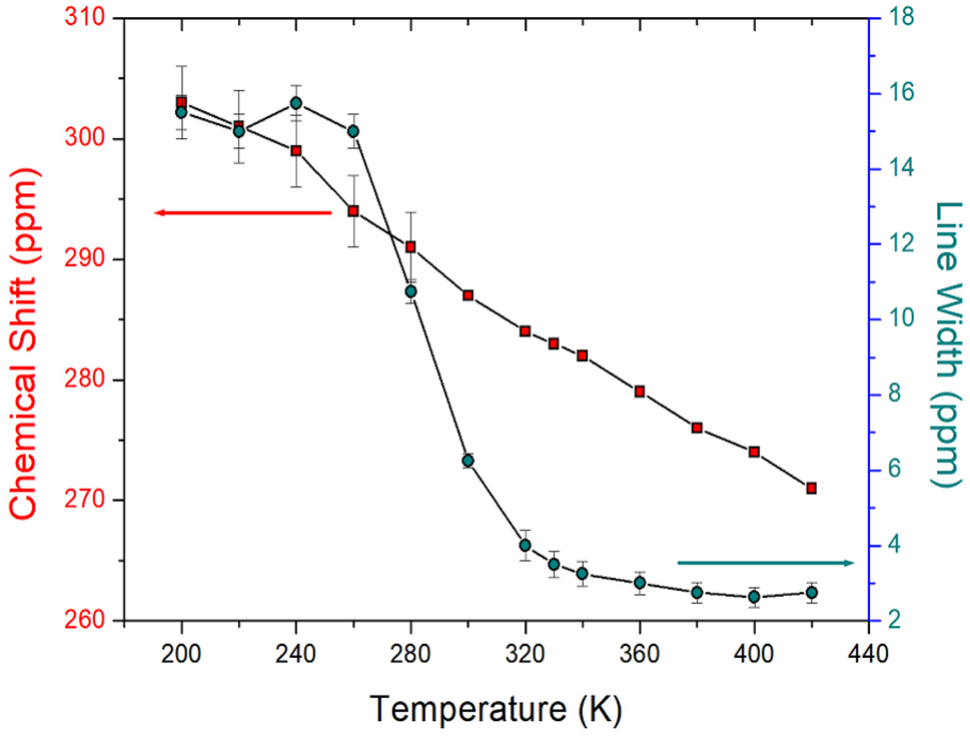
^113^Cd MAS NMR chemical shifts and line widths of [NH_3_(CH_2_)_2_NH_3_]_2_CdBr_4_·2Br single crystal as a function of temperature.

The chemical shift among the NMR parameters is very useful for clarifying the coordination environments around Cd^2+^ with unknown structures using ^113^Cd NMR spectroscopy^37^. In-situ ^113^Cd MAS NMR experiments, as a function of temperature, were employed to examine the structural environment in CdBr_6_ anions of [NH_3_(CH_2_)_2_NH_3_]_2_CdBr_6_, as shown in Fig. 9. NMR chemical shifts were recorded using CdCl_2_O_8_.6H_2_O as the standard. The sidebands of the ^113^Cd NMR signal are marked with asterisks as small signals located at equal intervals on both sides of the central peak. Unlike the ^1^H and ^13^C chemical shifts, the chemical shifts of ^113^Cd continuously decreased with increasing temperature from 200 to 420 K, as shown in Fig. 10. These are related to the change in the position of Br around Cd from the SCXRD results at 250, 300, and 350 K. The line width for ^113^Cd at 300 K is 6.23 ppm, and the ^113^Cd line width sharply narrows at temperatures above 260 K, which means that molecular motion becomes very active at high temperatures (Fig. 10). This result indicates that the environment of the Cd atom surrounded by Br atoms continuously changes with increasing temperature.

## Conclusion

We analyzed the crystal structures, phase transition temperatures, thermal behavior, and structural dynamics of the organic–inorganic hybrid [NH_3_(CH_2_)_2_NH_3_]_2_CdBr_6_, namely [NH_3_(CH_2_)_2_NH_3_]_2_CdBr_4_·2Br, crystals to investigate their physicochemical properties. Firstly, the monoclinic structure of this crystal was confirmed by single-crystal XRD, and the T_C_ of 443, 487, 517, and 529 K were determined using DSC and powder XRD results. This crystal had a good thermal stability of approximately 580 K, and mass loss was observed with increasing temperature owing to thermal decomposition, resulting in the loss of the 2HBr and 4HBr moieties. Secondly, the chemical shifts were caused by the local field around the resonating nucleus, and the ^1^H, ^13^C, ^14^N, and ^113^Cd NMR chemical shifts of the [NH_3_(CH_2_)_2_NH_3_] cation and CdBr_6_ anion varied with increasing temperature, suggesting that the surrounding environment changes with temperature; changes in the chemical shifts of ^1^H, ^13^C, ^14^N, and ^113^Cd were explained as affecting the coordination geometry of the hydrogen bond N‒H···Br connecting the cation and anion. From the SCXRD results, the position of Br around Cd was changed more than other atoms at 250, 300, and 350 K shown in CIF file, and the changes of the coordination geometry of Br around Cd in the CdBr_6_ and the coordination environments around N were related with changes in the hydrogen bond N‒H···Br. And, the displacement parameters of H bonded to N(1) in NH_3_ as shown in the supplementary data were the largest in relation to the change of the hydrogen bond N(1)-H-Br. In addition, ^1^H T_1ρ_ and ^13^C T_1ρ_, which represent the energy transfer around the ^1^H and ^13^C atoms of the cation, vary significantly with temperature. As show in Table 3, the E_a_ for molecular motion was found to be very high at ^13^C at high temperature compared to ^1^H. And, the fact that the line width of ^14^N is wider than those of ^1^H, ^13^C, and ^113^Cd means that the molecular motion of ^14^N is relatively rigid. This work serves to provide an understanding of these fundamental properties to broaden the application of organic– inorganic hybrid compounds.

## Data Availability

The datasets generated and/or analysed during the current study are available in the CCDC 2246614, 2246616, and 2246615. For ESI and crystallographic data in CIF or other electronic format see https://doi.org/.
